# Fast-Response and Reusable Oxytetracycline Colorimetric Strips Based on Nickel (II) Ions Immobilized Carboxymethylcellulose/Polyacrylonitrile Nanofibrous Membranes

**DOI:** 10.3390/ma11060962

**Published:** 2018-06-06

**Authors:** Mohammed Awad Abedalwafa, Yan Li, De Li, Xiaojun Lv, Lu Wang

**Affiliations:** 1 Key Laboratory of Textile Science and Technology, Ministry of Education, College of Textiles, Donghua University, Shanghai 200336, China; m.a.abedalwafa@uofg.edu.sd (M.A.A.); jwzhld@163.com (D.L.); tgy@dhu.edu.cn (X.L.); 2 Department of Technical Textile, Faculty of Industries Engineering and Technology, University of Gezira, Wad Madani 21111, Sudan

**Keywords:** nanofibrous membranes, nickel ions, colorimetric strips, oxytetracycline detection

## Abstract

Driven by economic interests, the abuse of antibiotics has become a significant concern for humans worldwide. As one of the most commonly used antibiotics, oxytetracycline (OTC) residue in animal-derived foods occurs occasionally, which has caused danger to humanity. However, there is still no simple and efficient solution to detect OTC residue. Here, an easily-operated colorimetric strategy for OTC detection was developed based on nickel ions (Ni^2+^) immobilized carboxymethylcellulose/polyacrylonitrile nanofibrous membranes (Ni@CMC/PAN NFMs). Owing to numerous O- and N-containing groups OTC has a strong tendency to complex with Ni^2+^ on the strips, inducing a color change from light green to yellow visible to the naked eye. The NFMs structural features, CMC functionalization process, and Ni^2+^ immobilization amount was carefully regulated to assure OTC detection whilst maintaining the inherent characteristics of NFMs. With the benefits of the large specific surface area (SSA) and small pore size of NFMs, the strips not only exhibited a rapid response (2 min), and low detection limit (5 nM) but also performed with good reversibility and selectivity concerning OTC detection over other antibiotics. The successful development of such enchanting nanofibrous materials may provide a new comprehension into the design and improvement of colorimetric strips.

## 1. Introduction

Oxytetracycline (OTC) is one of the tetracycline (TCs) antibiotics, which works by inhibiting bacterial protein synthesis in a broad range of Gram-positive and Gram-negative organisms [1]. According to the summary reports of Food and Drug Administration (USA Food & Drug Administration, 2015), TCs accounted for 38% of domestic sales of antimicrobials approved for use in food-producing animals for the year 2014 [2]. Because of its effective antimicrobial properties, good therapeutic effect, and relative inexpensiveness, OTC has become one of the most commonly used TCs in animal husbandry, aquaculture, and fruit crop production [3,4]. On the other hand, an exclusive drive for economic benefits results in OTC over-use, such that OTC residue in food products, such as meat, milk, eggs, honey, and fruits happens all the time [5,6,7]. Such residues might be transferred into the human body and result in liver damage, ototoxicity, nephrotoxicity, allergic reactions, and yellowish discoloration of teeth [8,9]. Worst of all, bacterial resistance to OTC in both humans and animals will occur [10,11,12,13,14], which has been highlighted by the World Health Organization (WHO) as a growing international concern. Hence, WHO recommended that the acceptable daily intake and the maximum residue limit of OTC are from 0–30 μg·Kg^−1^ (0–65 nM) body weight and 100 μg·Kg^−1^ (220 nM) respectively [15].

Up to now, to avoid humans suffering from the harm of food source OTC residues, different methods have been operated in practice, such as electrophoresis, high-performance liquid chromatography, electrochemical analysis, surface plasmon resonance, and microbiological techniques [16,17,18,19]. Nevertheless, these methods have faced some limitations as they are high-cost, time-consuming, have a low efficiency, and require well-trained persons [20]. Accordingly, the demand for an accurate, convenient, quick-response, and easily-operated method is the main motivation of the present study. Nowadays, visual colorimetric methods have received considerable attention because of their advantages, such as detection with the naked-eye, no requirement for sophisticated tools, easy-to-use, and relatively low cost [21]. Amongst the methods developed nowadays, colorimetric sensors, which set a target-induced naked-eye-visible color change as a signal, have become good candidates. In this case, researchers designed a liquid system for OTC colorimetric detection. For instance, OTC can perform as a reducer to synthesize gold nanoparticles, inducing a color change from yellow to pink [22]. Moreover, OTC can also complex with Fe_3_O_4_, leading to accelerated oxidation of tetramethylbenzidine by Fenton chemistry [16]. However, the naked-eye detection limit of systems mentioned above have still not fulfilled the requirement of the WHO, attributing to the drawbacks of the liquid system, which requires multiple-step dilution to be performed during detection. In addition, low shelf stability, non-portability, and long-term storage difficulties might also hinder their potential practical applications [23,24]. Consequently, there is considerable demand for developing a solid system for OTC detection.

Good sensors should have a small dimension, low fabrication cost, multiple functions, as well as high sensitivity, selectivity, and reliability. High sensitivity and fast responses require a sensor device with a large specific surface area (SSA) and a high porosity structure, which allow greater interaction with the target and improved sensitivity [25]. Electrospun nanofibrous membranes (NFMs) have some typical features like small pore size, high porosity, large SSA to volume ratio, excellent flexibility, and are easy to functionalize according to their various applications [26,27,28]. It is reasonable that the requirements of a good sensor can be fulfilled by applying nanofibrous material as a platform [29,30].

**Scheme 1:**
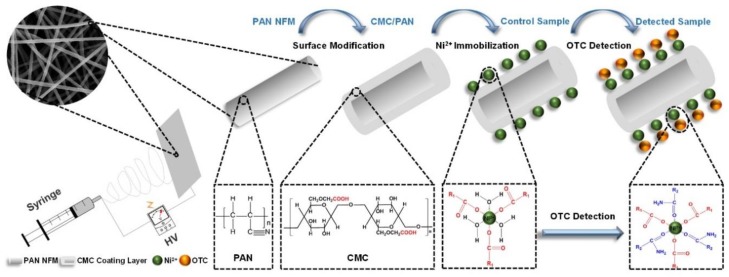
Schematic illustration of fabrication and detection concept of the Ni^2+^ immobilized CMC/PAN NFM colorimetric strips.

In the present study, colorimetric strips were developed for OTC detection based on composite NFMs; to the best of our knowledge no previous research has applied NFMs as a colorimetric platform to detect antibiotics. The designing protocol is illustrated in Scheme 1; firstly, Ni^2+^ was selected as a chromatic probe since it has the visual activity needed to complex with OTC through chemical coordination [31]. Secondly, the NFMs-based platform for Ni^2+^ immobilization was fabricated. Carboxymethylcellulose (CMC) is a crucial composite in the platform, with plenty of carboxymethyl groups in the molecular chain, which can serve as active sites for Ni^2+^ immobilization [32,33]. Unfortunately, the electrospinability of CMC is very poor. Thus, it cannot be fabricated directly into the platform [34]. On the other hand, electrospun polyacrylonitrile (PAN) NFMs presents good hydrophilicity, excellent chemical resistance, and great mechanical properties [35]. Therefore, PAN NFMs were selected as a support layer, then the platform of CMC/PAN was fabricated by modifying the surface of PAN NFMs with CMC. The optimum PAN, CMC, and Ni^2+^ concentrations were determined to yield the maximum color difference (ΔE) between the control sample and detected sample. Moreover, the colorimetric strips give a visible change in color from light-green to yellow based on the fact that immobilized Ni^2+^ can capture OTC in solution and form a Ni–OTC complex. Due to the strong-complex behavior between the colorimetric strips and OTC, the large SSA as well as the small pore size of the NFMs, the colorimetric strips showed a rapid response, high sensitivity, good selectivity, and is promising for real-work applications to detect OTC.

## 2. Materials and Methods

### 2.1. Materials and Reagents

PAN (M_w_ = 85, 000) and CMC (M_w_ = 300–800) were obtained from Shanghai Macklin Biochemical Co., Ltd., Shanghai, China. Nickel chloride hexahydrate (NiCl_2_·6H_2_O) was received from Shanghai Titan Scientific Co., Ltd., Shanghai, China. *N*,*N*-dimethylformamide (DMF), sodium hydroxide (NaOH), and hydrochloric acid (HCl) were purchased from Shanghai Lingfeng Chemical Reagent Co., Ltd., Shanghai, China. OTC was brought from Adamas Reagent Co., Ltd. (Shanghai, China). Sulfadiazine (SDZ), Chloramphenicol (CAM), and Gentamicin (GEN) were obtained from Aladdin Industrial Corporation Shanghai (Shanghai, China). While Norfloxacin (NRFX), Penicillin G (PC-G), Azithromycin (AZM), and Cephalexin (CEF) were obtained from Songon Biotech Shanghai Co., Ltd. (Shanghai, China). All chemicals were used as received.

### 2.2. Preparation of PAN NFMs

PAN NFMs were fabricated by applying the electrospun technique. Firstly, PAN was dried at 95 °C in vacuum for 2 h, then dissolved in DMF and stirred at room temperature for 24 h. The homogeneous solutions were electrospun using the optimized parameters of 1 mL/h flow rate, 20 kV applied voltage, a 15 cm working distance, and 19 G needle until the thickness of the PAN NFMs reached to around 70 μm. The electrospun chamber was kept at a constant temperature of 28 °C and relative humidity of 30%. The NFMs were dried in vacuum oven at 70 °C for 1 h. The concentrations of PAN were adjusted to 8, 10, 12, and 15 wt % (PAN_8_, PAN_10_, PAN_12,_ and PAN_15_), to study the effect of PAN concentrations on colorimetric strips performance.

### 2.3. Surface Modification of PAN NFMs

The surface modification of PAN NFMs with CMC was performed according to the method used by Ahire et al. [36]. In brief, PAN_x_ NFMs with different concentrations were cut into 10 mm × 10 mm pieces with an average thickness of 70 ± 5 μm. Then PAN_x_ NFMs were soaked separately into a series of CMC_y_ aqueous solutions, which have different concentration of 0.1, 0.3, 0.5, 0.7, 0.9, and 1.1 wt % (CMC_0.1_, CMC_0.3_, CMC_0.5_, CMC_0.7_, CMC_0.9_, and CMC_1.1_) for 72 h to study the influence of CMC on strips performance. Subsequently, the CMC modified NFMs were washed with distilled water for several times to remove the CMC residue. Finally, CMC_y_/PAN_x_ NFMs were dried at room temperature for 24 h before use.

### 2.4. Fabrication of Colorimetric Strips

The Ni_z_@CMC_y_/PAN_x_ NFMs colorimetric strips were fabricated by immobilization of Ni^2+^ on the surface of CMC_y_/PAN_x_ NFMs. Typically, the as-prepared CMC_y_/PAN_x_ NFMs were immersed into an aqueous solution of nickel chloride for 6 h under pH of 5, then dried at room temperature. The unreacted ions were washed from the nanofibrous material with distilled water and dried again. Finally, the effect of Ni^2+^ concentrations was investigated by utilizing a various concentration of 0.01, 0.1, and 1 M (Ni_0.01_, Ni_0.1,_ and Ni_1_).

### 2.5. Detection of OTC

The created colorimetric strips were exposed to OTC solutions for 30 min at variable pHs: 2, 3, 4, 5, 6, 7 or 8 at room temperature to study the effect of pH on OTC detection. The strips were subsequently removed from the solution, then washed with 5 mL distilled water and dried at room temperature. Further, the effect of time on OTC detection was studied by exposing the strips to 10 mM of OTC solutions for various times (0 to 30 min) at room temperature. Moreover, the influence of temperature on OTC detection was investigated by exposing the strips to 10 mM of OTC solutions for 30 min at various temperatures (10 to 90 °C). Furthermore, to explore the sensitivity of the colorimetric strips to OTC, the strips were exposed to OTC in various concentrations (0 to 5 mM) at the optimized conditions. A similar procedure was also applied for the detection of different antibiotics in solutions NRFX, SDZ, CAM, PC-G, CEF, AZM, and GEN.

### 2.6. Characterization

Field emission scanning electron microscopy (FE-SEM, S-4800, Hitachi Ltd., Tokyo, Japan), selected area electron diffraction (SAED), and energy dispersive X-ray spectroscopy (EDX, JEM-2100F, JEOL Ltd., Akishima, Tokyo, Japan) were employed to observe the microstructure and composition of the obtained NFMs. The FE-SEM images were analyzed using Adobe Acrobat software to get the diameter. The N_2_ adsorption-desorption analyzer (ASAP2020, Micromeritics Co., Norcross, GA, USA) was used to characterize the porous structure and surface area of NFMs. The capillary flow porometer (CFP-1100AI, Porous Materials Inc., Ithaca, NY, USA) was used to analyze pore size distribution of NFMs. The NFMs were analyzed by Fourier transform infrared-attenuated total reflectance (FTIR-ATR) spectroscopy, (Nicolet 6700 FTIR spectrometer, IET Ltd., Mundelein, IL, USA). An IS-30-6-R integrating sphere (Ideaoptics Technology Ltd., Shenzhen, China) attached to an Ideaoptics PG 2000+ fiber optic spectrometer was applied to measure the reflectance (%R) of the strips, and the test was repeated and analyzed to ensure consistency. The corresponding absorbance was calculated by using Schuster–Kubelka–Munk equation at any wavelength [37], F (%R) = (1 − %R)^2^/2 × %R. The color difference (ΔE) between two colors is determined as the Euclidean distance between these two sets of color coordinates, which was calculated as: ΔE^2^ = (*L*_1_ − *L*_2_)^2^ + (a_1_ − a_2_)^2^ + (b_1_ − b_2_)^2^, and a value of ΔE = 1 is approximately equal to the threshold of perceptual color change [38]. The coating ratio was calculated by using the equation: coating ratio (%) = [(W_CMC/PAN_ − W_PAN_)/W_PAN_] × 100, where, W_CMC/PAN_ represents the weight of PAN NFMs after coating and W_PAN_ weight before CMC coating. All the results were averaged from five replicates and presented as the average ± the standard deviation.

## 3. Results and Discussion

### 3.1. Fabrication of Colorimetric Strips

**Figure 1:**
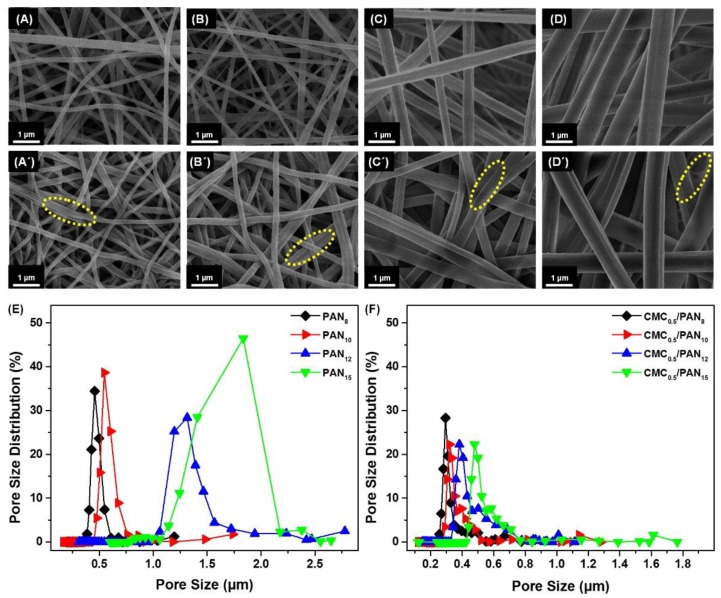
FE-SEM images of (**A**–**D**) electrospun PAN NFMs of different concentration 8, 10, 12 and 15 wt % respectively. (**A′**–**D′**) the corresponding NFMs coated with 0.5 wt % CMC respectively. Their corresponding pore size distribution curves before and after the coating shown in (**E**,**F**).

To design such sensitive and selective colorimetric strips needed to trace OTC requires immobilizing of a colorimetric agent into a porous substrate with good homogeneity and stability. As illustrated in Scheme 1, our colorimetric strips were mainly based on Ni^2+^ immobilized CMC/PAN NFMs. Different strips obtained from different PAN concentrations (PAN_8_, PAN_10_, PAN_12_, and PAN_15_) were fabricated to investigate the effect of the electrospun solution concentrations on the structure and morphology of the NFMs as well as the performance of the colorimetric strips. The representative FE-SEM images of the PAN_x_ NFMs with various concentrations displayed a randomly oriented three dimensional (3D) nonwoven structure which is illustrated in Figure 1A–D. It is evident that all fabricated NFMs presented a reasonably good regularity. Interestingly, the average diameter of PAN NFMs increased from 127 to 716 nm for increasing concentration of the solution from 8 to 15 wt %, as demonstrated in Figure S1A–D. The increasing tendency of the average nanofiber diameters depended on the increase in the viscosity of the electrospun solution [39]. Besides, after coating with CMC_0.5_, the average diameter of nanofibers after post-processing increased in range from 135 to 822 nm. Meanwhile, the adhesion structures between nanofibers could be observed among adjacent fibers, as indicated in Figure 1A′–D′ (indicated by a dotted yellow circle). This result is related to the coating and the drying process of the NFMs [36].

**Figure 2:**
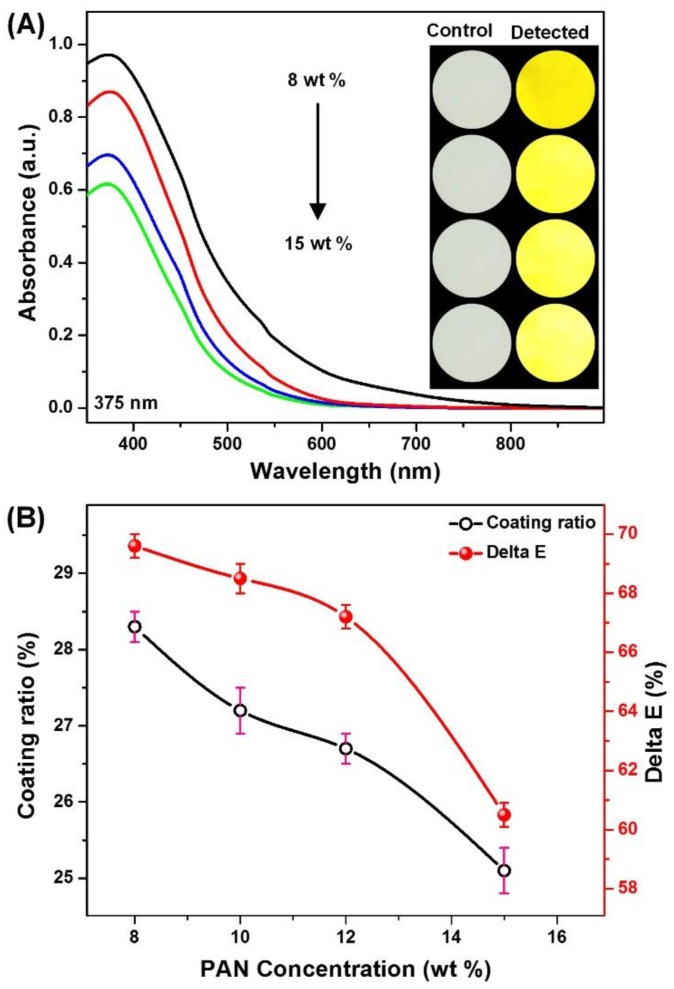
(**A**) The absorption spectra showing the effect of PAN concentration in colorimetric strips response after incubation for 30 min in the presence of OTC (10 mM); and (**B**) the maximum absorption and the color difference values as a function of PAN concentration; the corresponding optical images are shown as inserts.

Based on the morphological investigation of the PAN*_x_* NFMs before and after coating by 0.5 wt % CMC, the pore size distribution of the NFMs was also studied; as shown in Figure 1E, the uncoated NFMs showed a pore size distribution ranging from 0.1 to 2.8 µm. The maximum pore size of the NFMs increased from 1.19 to 2.78 μm along with increasing the concentration of the electrospun solutions. By comparing all the PAN*_x_* NFMs, PAN_8_ displayed relatively smaller and more uniform pore sizes than the others, which is consistent with the previously reported case [40]. Moreover, after coating the NFMs with 0.5 wt % CMC, the maximum pore size decreased for all the samples with pore size distribution ranging from 0.1 to 1.8 µm, as can be seen from Figure 1F, which resulted from the adhesive structure. Although CMC_0.5_/PAN_8_ had a relatively small and uniform pore size in comparison with the other three samples, it achieved the highest coating ratio (see Figure 2B and Table S1), which may be attributed to their small fiber diameter [41]. Therefore, smaller pore size structure and high coating ratio could be observed in CMC_0.5_/PAN_8_, which provided numerous small microporous channels that could significantly boost the Ni^2+^ adsorption [42], which is beneficial for enhancing the detection performance.

Subsequently, the Ni_0.1_@CMC_0.5_/PAN*_x_* NFMs were exposed to 10 mM OTC for 30 min, and the absorption spectra were spotted, as shown in Figure 2A,B. Indeed, the absorbance intensity at 375 nm and the color difference of Ni_0.1_@CMC_0.5_/PAN_8_ NFMs are higher than the other three samples. In principle, the smaller pore size could facilitate OTC diffusion into the NFMs, as well as Ni^2+^ adsorption [43]. This result presented the significant contributing role of PAN concentration in the improvement of the detection performance of colorimetric strips, which is consistent with the FE-SEM and pore size analysis.

**Figure 3:**
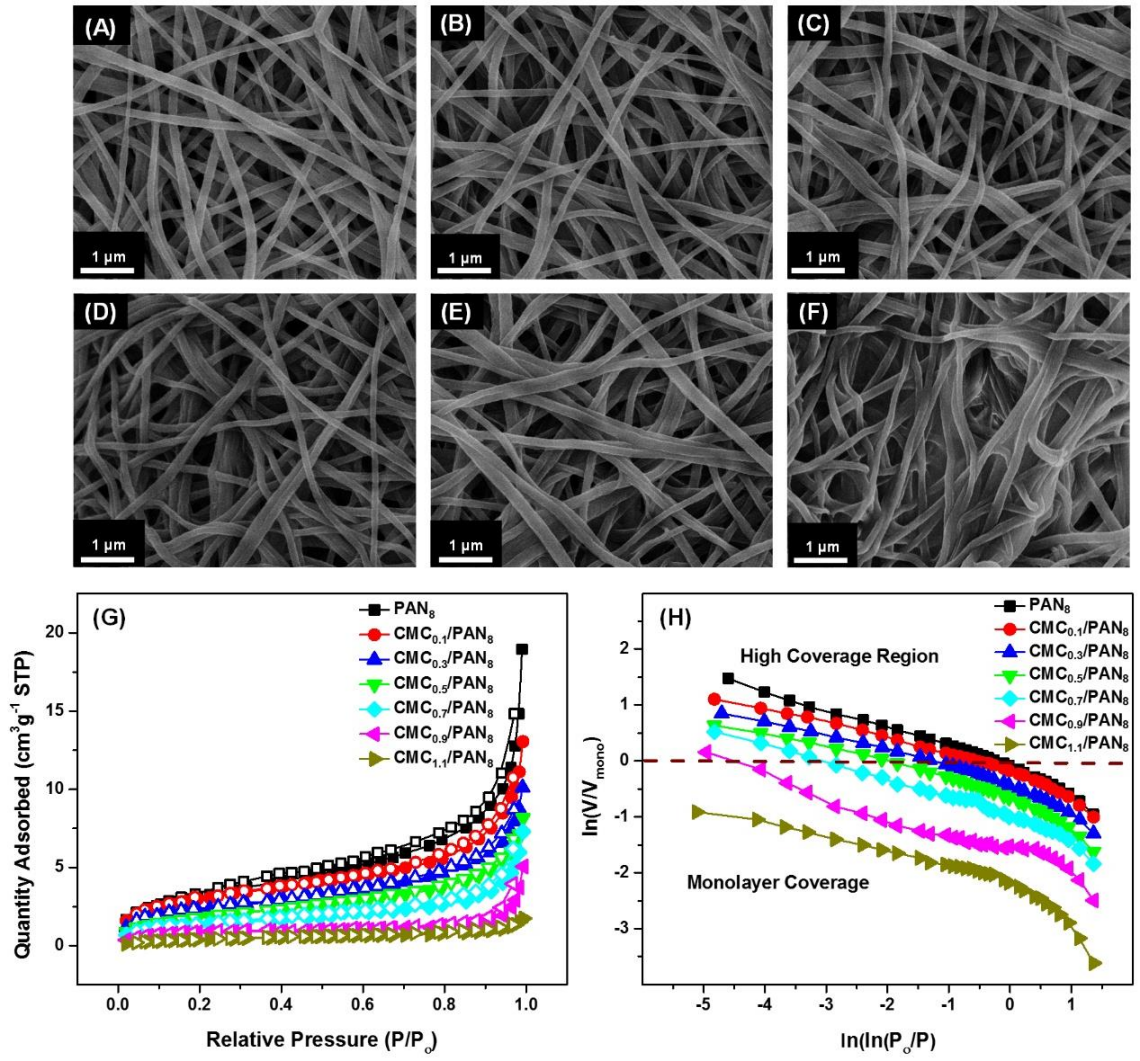
FE-SEM images of the PAN_8_ NFMs coated with various CMC concentrations of (**A**) 0.1; (**B**) 0.3; (**C**) 0.5; (**D**) 0.7; (**E**) 0.9; and (**F**) 1.1 wt %; (**G**) Nitrogen adsorption-desorption isotherms of the PAN_8_ NFMs and the corresponding coated NFMs (**H**) the corresponding FHH plot of ln(V/V_mono_) against ln(ln(P_0_/P)) reconstructed from (G).

The above results have demonstrated that the carbonyl groups on CMC were the key factor for Ni^2+^ adsorption process. Thus, to further improve the limit of detection of OTC, the CMC concentration to modify the PAN_x_ NFMs needs to be optimized. PAN_8_ NFMs were selected to appreciate the influence of CMC concentration on the sensitivity of the colorimetric strips. The representative FE-SEM images of the CMC_y_/PAN_8_ NFMs acquired by altering CMC concentrations are shown in Figure 3A–F, indicating that the nanofibers evidently were adhered and connected to form “adhesive structures”. Additionally, with increasing concentration of CMC from 0.1 to 1.1 wt %, the area of the adhesive structure extended and the nanofibrous fiber diameter increased from 131 to 152 nm, as well as the coating ratio (Table S2) [40]. This phenomenon was attributed to the formation of a thin CMC coating layer which increased with increasing CMC concentrations [36]. Therefore, a more adhesive structure appeared along with increasing concentrations of CMC.

On the other hand, considering that colorimetric detection performance highly relies on SSA, the effect of the various CMC concentrations on the SSA of the CMC_y_/PAN_8_ NFMs was systematically investigated via the N_2_ adsorption-desorption method. As shown in Table S3, the relevant Brunauer–Emmett–Teller (BET) surface areas revealed a remarkable decrease from 11.39 to 1.479 m^2^·g^−1^, and the pore volume decreased from 0.02931 to 0.00269 cm^3^·g^−1^ accordingly, which is due to the increase of CMC concentrations causing the adhesion structure among the nanofibers, blocking their porous structure. Figure 3G shows the isotherms were of type IV according to the International Union of Pure and Applied Chemistry (IUPAC) classification with H3 hysteresis loop, which demonstrates that the macro-porous structure existed within the as-prepared CMC_y_/PAN_8_ NFMs [44,45]. Moreover, the hierarchical textures of the as-prepared CMC_y_/PAN_8_ NFMs were further quantified through fractal analysis according to the Frenkel–Halsey–Hill (FHH) theory, which is calculated on the basis of the nitrogen adsorption isotherm [46]. Figure 3H presents the corresponding FHH plots of the relevant CMC_y_/PAN_8_ NFMs, in which the straight-line slopes located in the high coverage region were nearly the same. The calculated fractal dimension (D) was 2.64, 2.68, 2.67, 2.66, 2.66, 2.67, and 2.66, respectively, which further verifies that the porous structures of as-prepared membranes were chiefly composed of macro-pores. These results represent the main contributing role of CMC in deciding the surface area of NFMs, which is consistent with the FE-SEM analysis, as mentioned above.

**Figure 4:**
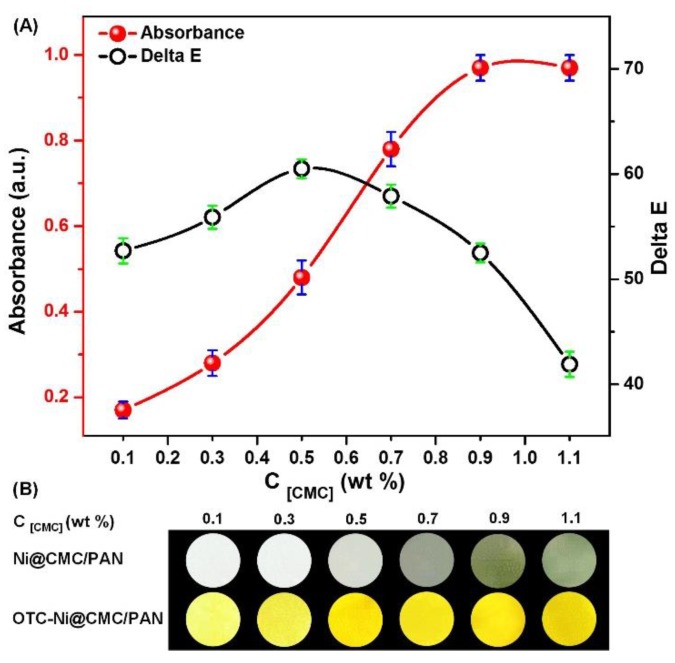
(**A**) The maximum absorbance and color difference values of the colorimetric strips after incubation for 30 min in the presence of OTC (10 mM) as a function of CMC concentrations; and (**B**) the corresponding optical images before and after incubation.

Posteriorly, the Ni_0.1_@CMC_y_/PAN_8_ NFMs with different CMC concentrations were exposed to 10 mM OTC for 30 min, and the absorbance was observed, as displayed in Figure 4A. It is apparent that the absorbance intensity gradually increased with the increase of CMC concentrations; when the coating percentage of CMC was 1.1 wt % (CMC_1.1_) the absorption intensity reached a maximum value. This may be ascribed to the increasing number of active sites for Ni^2+^ immobilization on the NFMs, thereby the OTC detection ability was theoretically improved. However, the calculated color difference values between the OTC-reacted and control samples indicated that CMC_1.1_ was not an optimum coating concentration. As shown in Figure 4B, PAN_8_ NFMs coating with CMC_0.5_ displayed the most significant color differences. Referring to the photograph of each sample shown in Figure 4B, it was found that the Ni^2+^ adsorption capacity of the NFMs coating with 1.1 wt % was higher than the others. Therefore, the color of 1.1 wt % coated NFMs displayed a deep green color, due to Ni^2+^ adsorbed on the surface of the NFMs resulting from low SSA (1.479 m^2^·g^−1^). The liquor waited for detection contained only tiny amounts of OTC, correspondingly causing color changes which were covered up by the background color (deep green). As a result, it was concluded that CMC_0.5_ is the optimum coating concentration for our detection. This is attributable to the relatively high SSA, which is 4.4 times higher than CMC_1.1_.

To further comprehend the relation between colorimetric response and immobilizing amount of Ni^2+^, a series of CMC_0.5_/PAN_8_ NFMs immobilized with different concentrations of Ni_0.01_, Ni_0.1_ and Ni_1_ were fabricated. Figure S2A provides a comparison of the absorbance intensity at 375 nm of the OTC detection (10 mM) by the CMC_0.5_/PAN_8_ NFMs immobilized with three different Ni^2+^ concentrations. It clearly shows that there is no significant difference between the samples regarding the absorbance intensity at 375 nm, which is likely due to the colorimetric reaction reaching saturation resulting from the same amount of OTC. Nevertheless, the strips immobilized with Ni_0.1_ was selected as the optimum immobilizing amount for OTC detection. This is because it had the most significant color difference value, as displayed in Figures S2B and S3, which is probably due to the increased immobilized amount of Ni^2+^ with increasing concentration appearing as a more in-depth green color of the control sample.

**Figure 5:**
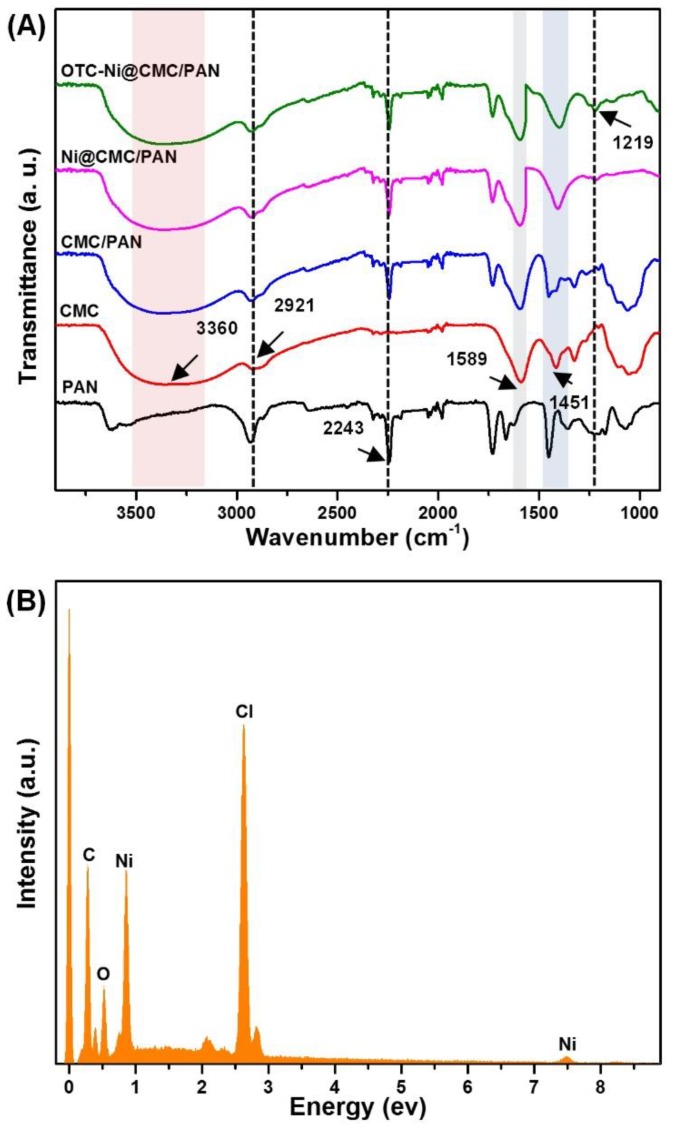
(**A**) FTIR spectra of NFM in the various fabrication stages of the strips. (**B**) EDX spectrum of Ni@CMC/PAN NFM.

Generally, Ni^2+^ has a visual response to OTC in aqueous solution [31]. Additionally, since PAN NFMs cannot absorb Ni^2+^, a surface modification would be desired for Ni^2+^ immobilization. The incapable Ni^2+^ absorbing ability of PAN NFMs has been proven in Figure S4; the white color of PAN NFM was maintained after exposure in Ni^2+^ solution for 6 h. Subsequently, with the aim of endowing the PAN NFM with Ni^2+^ adsorption capacity, the NFMs were further functionalized with CMC. Evidence for the functionalization of the PAN NFM with CMC came from the FT-IR spectral analysis. As displayed in Figure 5A, characteristic peaks around 3360 cm^−1^, 2921 cm^−1^, and 1589 cm^−1^ in the spectrum of the CMC were assigned to the stretching vibration of -OH, CH, and COOH respectively. The peak around 2243 cm^−1^ in the spectrum of the PAN, belonged to the C≡N. It is worthwhile noting that all the peaks of the CMC/PAN NFM belonged to PAN and CMC, which indicates the successful coating of CMC on the PAN NFM. The success of the functionalization process is due to the strong hydrogen bond between aliphatic hydroxyl (C-OH) in CMC and the cyano group (C≡N) in PAN NFM [47,48,49]. These results suggest that the PAN NFM has been successfully modified with CMC and the hydroxyls are preserved. Because of the strong hydrogen bond, the CMC is formed within the PAN NFM, thus improving its stability and preventing it from leaking out of the PAN NFM.

Nonetheless, the color of the functionalized CMC/PAN also persisted without noticeable changes when exposed to OTC solution. Therefore, Ni^2+^ as a colorimetric agent has been immobilized on the CMC/PAN NFM. As shown in the FTIR results in Figure S5A, after Ni^2+^ immunization (Ni@CMC/PAN NFM), the peaks at 1595 cm^−1^ and 1451 cm^−1^ were shifted to 1590 cm^−1^ and 1404 cm^−1^ respectively. Here, results indicate that the Ni^2+^ attached to the carboxyl groups and certain chemical bonds were formed (unidentate coordination) [50,51], which caused a decrease in the vibration frequency of these surface chemical groups, see Figure S5B. Additionally, as indicated in Figure 5B, the corresponding EDX spectrum further confirmed the co-existence of Ni element in the CMC/PAN NFM.

UV-vis absorption spectroscopy was used to study the interaction of colorimetric strips with OTC. As shown in Figure S4, after the incubation of the colorimetric strips in OTC solution (10 mM) for 30 min, the color changed from light-green to yellow and the absorption peaks at 735 nm shifted to 375 nm, which indicated the interaction between the colorimetric strips and OTC. In addition, as can be seen from Figure 5A, a new peak appeared around 1219 cm^−1^ in the spectrum of the OTC-Ni@CMC/PAN which belonged to the C-N, which also indicated the interaction between the colorimetric strips and OTC [52]. We believe the color change is due to the oxidation of the OTC through the binding of the carbonyl oxygen atom at the amide group to the center of Ni^2+^, as indicated in Figure S5C [53,54].

### 3.2. The Optimization of Detection Conditions

For the sake of improving the sensitivity of the established colorimetric strips, the experimental conditions for OTC detection were carefully optimized at first. As described above, the proposed rapid detection of OTC was based on the spectral and color changes of the colorimetric strips caused by the interaction between the Ni^2+^ and the OTC. Therefore, the performance of this detection would be strongly influenced by the detection conditions, including pH of the OTC solution, incubation time, and temperature.

**Figure 6:**
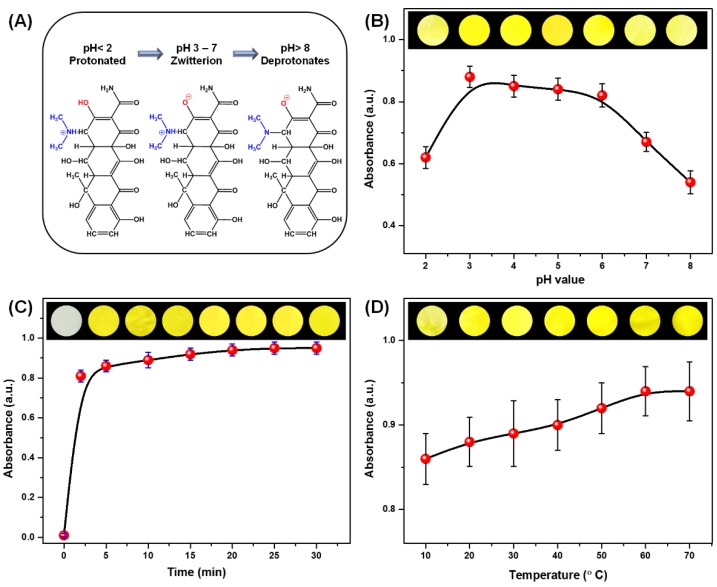
(**A**) The effect of the pH on the chemical structure of OTC. Optimization of OTC detection conditions: the maximum absorbance intensity with the change of (**B**) pH, (**C**) time, and (**D**) temperature; the corresponding optical images are shown as inserts.

Firstly, to explore the optimum pH of OTC solution, different pH conditions (rangeing from 2 to 8) were performed to understand the influence of pH condition on the sensitivity of colorimetric strips. At a pH greater than 8, nitrogen electrons of dimethylamine group compete with the oxygen of the amide group for the binding of metal ions, which will lead to diminishing interaction speeds, see Figure 6A [55]; the solubility of OTC also appears to decrease [56]. After exposing colorimetric strips with 10 mM OTC under different pH conditions for 30 min, the spectral absorption characteristics were shown in Figure 6B. As can be seen, the absorbance at 375 nm rapidly increased as the pH condition raised to 3 and then persisted constantly between pH 3 to 6, after which, a straight decrease in absorbance was observed at pH greater than 6. The Ni-OTC complex exhibits maximum absorbance at 375 nm when pH adjusts to 3. The OTC-Ni compound is found to be highly stable in this condition, due to the singlet oxygen formation rate decreasing with increasing pH. A media of pH lower than 2 is not recommended as the OTC is not stable and Ni^2+^ can be removed from the strips under highly acidic conditions. The absorbance values displayed a decrease when the pH was higher than 6, which may occur due to the possible dissociation of the complex [57]. Thus, pH 3 was selected as the optimum pH value for OTC detection.

Subsequently, to increase the sensitivity for the detection of OTC, the kinetic sensing response was studied, since reaction time is an important parameter to be optimized in analytic assays. Ten millimolar OTC liquor was prepared, and the incubation times were set at intervals of 0, 2, 5, 10, 15, 20, 25, and 30 min. It can be seen clearly from Figure 6C that an obvious tendency of absorbance intensity at 375 nm showed a slight increase with increasing the incubation time. Even if the incubation time was prolonged, the observed results showed that the absorbance intensity at 375 nm had shown a small increase, and there seemed to be no visible color changes according to the digital photograph shown in Figure 6C.

Finally, to further understanding of the sensing performance of the colorimetric strips, the effect of the temperature on colorimetric detection was studied; fundamental knowledge on such exposure is significant for OTC [58]. The relationship between detection temperatures in the range of 10 °C to 70 °C and the absorbance intensity at 375 nm is shown in Figure 6D. Temperature during the detection can be up to 70 °C, so 70 °C was chosen as the maximum temperature in the experiments. As illustrated in Figure 6D, the temperature-dependent absorbance intensity at 375 nm shows a stable range from 20 to 70 °C. Notably, rising of the temperature to 20 °C could guarantee the saturation of the concentration of Ni^2+^ on the membranes, since further increasing the temperature could not lead to a dramatic intensity change.

### 3.3. The Sensitivity of Colorimetric Strips

**Figure 7:**
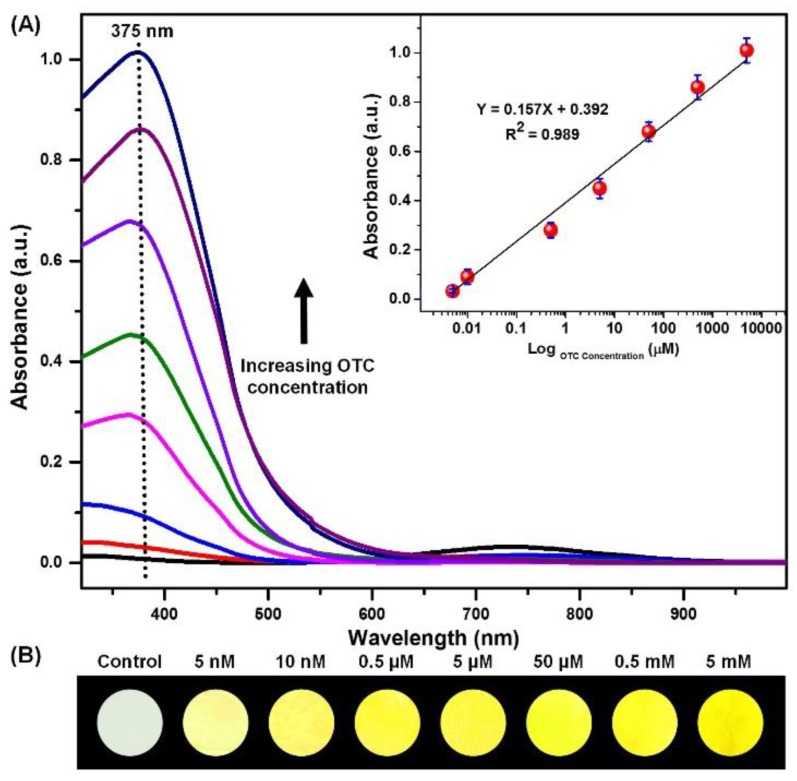
(**A**) The absorption spectra and (**B**) the corresponding optical images of colorimetric strips upon exposure to OTC at different concentrations (0 to 5 mM) at optimal conditions; the correction linear relation graph is shown as an insert.

To evaluate the limits of detection of these colorimetric strips, a series of OTC liquor with concentrations from 0 to 5 mM were adjusted into optimal conditions, and the absorption spectra were detected after 2 min of detection. In Figure 7A, it is clear that with increasing OTC concentrations from 0 to 5 mM, the absorbance intensity at 375 nm increased progressively. Also, monitoring of the corresponding optical images of the strips indicated that OTC could lead to a visible color change from light-green to yellow; even at low concentration of 5 nM the induced color change was easy to be noticed by naked-eye, as can be evidenced in Figure 7B. Moreover, a good linear relationship between maximum absorbance at 375 nm and Log OTC concentrations ranging between 0 and 5 mM was also observed with R^2^ value of 0.9863. This indicates that color change is proportional to the amount of OTC and forms the fundamental principle to quantify the OTC amount using the colorimetric strips. Furthermore, it is worth mentioning that a significantly low concentration of OTC (5 nM) could be detected by the naked-eye, which was imputable to the large SSA, the homogeneous immobilization of Ni^2+^ on the NFMs, the strong-complex behavior, and the small pore size of the NFM.

### 3.4. Selectivity and Reversibility of Colorimetric Strips

**Figure 8:**
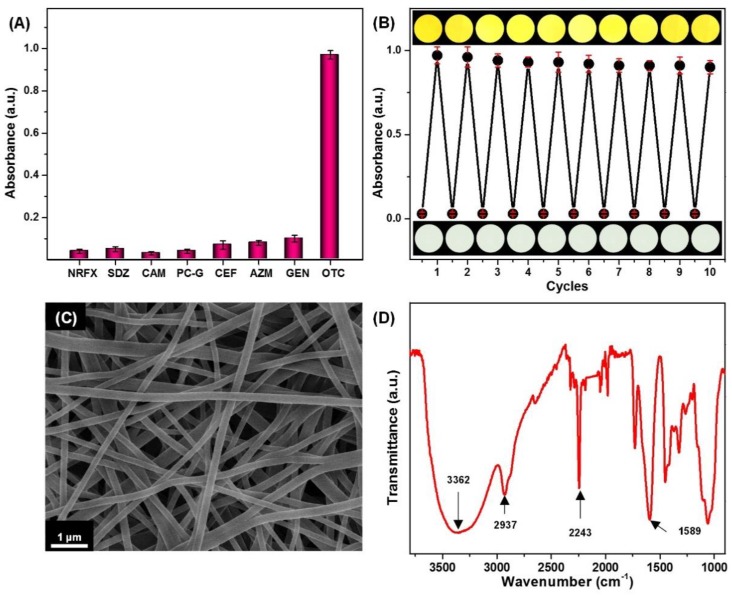
(**A**) The absorption intensity of the colorimetric strips indicating the selectivity after incubation for 20 min in the presence of OTC and various antibiotics at a concentration of 10 mM; (**B**) the absorbance intensity and optical colorimetric response, showing the reversibility of the colorimetric strips; (**C**) SEM image; and (**D**) FT-IR spectra of the colorimetric strips after 10 regeneration/reuse cycles.

The selectivity of a sensing system is an essential feature when these strips are expected to be applied to real-world applications. Therefore, a consistent investigation about the selectivity of the colorimetric strips by exposing them to various antibiotic solutions including NRFX, SDZ, CAM, PC-G, CEF, AZM, and GEN was done. As detailed in Figure 8A, only OTC resulted in a remarkable absorbance increase at 375 nm and a visible color change from light-green to yellow, indicating that the other antibiotic solutions have a modest impact because of the specific reaction between OTC and the colorimetric strips. As mentioned in Figure 8A, 10 mM of OTC caused a remarkable color change, while most of the other antibiotics showed mostly invisible color changes. That is because OTC has an oxygen of the amide group adjacent to the hydroxyl group; the color variations can be attributed to the complexation of OTC with Ni^2+^ through oxygen atoms in coordination [52]. Notably, the new colorimetric strips are promising for commercial applications to detect OTC.

Lastly, to achieve excellent reversibility, decolorizing procedures were introduced to regenerate the activity of the colorimetric strips. Within each cycle, the colorimetric strips were treated with 0.1 wt % hydrogen chloride solution for 10 min then rinsed with distilled water, followed by drying at room temperature. Thus, the color of the strips would switch to white again. For a second time, the strips were immersed into 0.1 M solution of nickel chloride for 6 h to be reused for another cycle. Notably, the absorption spectra studies showed that the regenerated strips retained more than 92% of the original absorption intensity after 10 regeneration/reuse cycles (Figure 8B), which indicated the successful chemical removal of Ni-OTC components and the formation of activated colorimetric strips. Such excellent reversibility for OTC detection could be due to: (1) as indicated in Figure 8C, the durable stability of the unique snaky interconnected fibrous structure that prevents the PAN NFM from breaking up after repeated use; (2) the homogenous and the stable CMC modification layers throughout its tortuous porous structure, as indicated in Figure 8D. It is worthwhile to note the FTIR spectrum of CMC/PAN in Figure 5A; their characteristic peaks can also be obeserved in Figure 8D. These results indicate the CMC coating layer still remains on the PAN NFM surface because of the strong hydrogen bond.

## 4. Conclusions

In summary, we have established a strategy for designing and fabricating colorimetric strips based on Ni@CMC/PAN NFM, by a combination of the electrospun technique, surface modification, and immobilization, for OTC detection. Because of the strong-complex behavior between Ni^2+^ and OTC, which caused the color change, the visual colorimetric strips for OTC detection attained based on the NFM. Benefiting from the large SSA, high pore volume, and small pore size of the NFM, the colorimetric strips achieved a fast response within 2 min and an evident naked-eye detection limit of 5 nM. Moreover, the colorimetric responses showed good linearity in the concentration range from 5 nM to 5 mM. Furthermore, the colorimetric strips exhibited excellent selectivity to OTC over other antibiotics and good reversibility after ten cycles. The successful fabrication of such enchanting nanofibrous materials by using this simple strategy may offer a model on the design and development of colorimetric material.

## Supplementary Materials

The following are available online at http://www.mdpi.com/1996-1944/11/6/962/s1, Figure S1: The diameter distributions of electrospun PAN NFMs of different concentration (A) 8 wt %, (B) 10 wt %, (C) 12 wt % and (D) 15 wt %., Figure S2: (A) The absorption spectra, and (B) the color difference values between the control and detected samples of the colorimetric strips as a function of Ni^2+^ concentration; the corresponding optical images are shown as inserts, Figure S3: (A) The absorption spectra, and (B) the corresponding optical images of the CMC/PAN NFM upon exposure to Ni^2+^ solution at different concentrations (0.01, 0.1 and 1 M), Figure S4: The color response of different NFM after exposure to 10 mM OTC solution for 30 min, Figure S5: (A) FTIR spectra of CMC coated PAN FNM before and after Ni^2+^ immobilization and (B) the coordination mode of CMC with Ni^2+^, Table S1: The coating ratio of PAN NFM coated by 0.5 wt % CMC, Table S2: The Coating ratio of 8 wt % PAN NFM coated CMC with different concentration, Table S3: BET surface areas and total pore volume of the PAN_8_ NFMs coated with various CMC concentrations (0, 0.1, 0.3, 0.5, 0.7, 0.9, and 1.1 wt %).
