# Supplementary material for: Fast-Response and Reusable Oxytetracycline Colorimetric Strips Based on Nickel (II) Ions Immobilized Carboxymethylcellulose/Polyacrylonitrile Nanofibrous Membranes
Source: Materials (Basel). 2018 Jun 6;11(6):962. doi: 10.3390/ma11060962 (PMC6025156; doi:10.3390/ma11060962)
Supplement: Supplementary file 1 [file materials-11-00962-s001.pdf]

materials

Supplementary Materials:

Fast-Response and Reusable Oxytetracycline Colorimetric Strips Based on Nickel (II) Ions Immobilized Carboxymethylcellulose/ Polyacrylonitrile Nanofibrous Membranes

Mohammed Awad Abedalwafa [1,2], Yan Li [1,*], De Li [1], Xiaojun Lv [1] and Lu Wang [1,*]

[1] Key Laboratory of Textile Science and Technology, Ministry of Education, College of Textiles, Donghua University, Shanghai 200336, China; m.a.abedalwafa@uofg.edu.sd (M.A.A.); jwzhld@163.com (D.L.); tgy@dhu.edu.cn (X.L.)

[2] Department of Technical Textile, Faculty of Industries Engineering and Technology, University of Gezira, Wad Madani 21111, Sudan

* Correspondence: yanli@dhu.edu.cn (Y.L.); wanglu@dhu.edu.cn (L.W.); Tel.: +86-21-6779-2634 (Y.L.); Fax: +86-21-6779-2637 (L.W.)

Supplementary Materials: The following are available online at www.mdpi.com/link, Figure S1: The diameter distributions of electrospun PAN NFMs of different concentration (A) 8wt%, (B) 10wt%, (C) 12 wt% and (D) 15 wt%., Figure S2: (A) The absorption spectra, and (B) the color difference values between the control and detected samples of the colorimetric strips as a function of Ni^{2+} concentration; the corresponding optical images are shown as inserts, Figure S3: (A) The absorption spectra, and (B) the corresponding optical images of the CMC/PAN NFM upon exposure to Ni^{2+} solution at different concentrations (0.01, 0.1 and 1 M), Figure S4: The color response of different NFM after exposure to 10 mM OTC solution for 30 min, Figure S5: (A) FTIR spectra of CMC coated PAN FNM before and after Ni^{2+} immobilization and (B) the coordination mode of CMC with Ni^{2+}, Table S1: The coating ratio of PAN NFM coated by 0.5 wt% CMC, Table S2: The Coating ratio of 8 wt% PAN NFM coated CMC with different concentration, and Table S3: BET surface areas and total pore volume of the PAN_8 NFMs coated with various CMC concentration (0, 0.1, 0.3, 0.5, 0.7, 0.9, and 1.1 wt%)

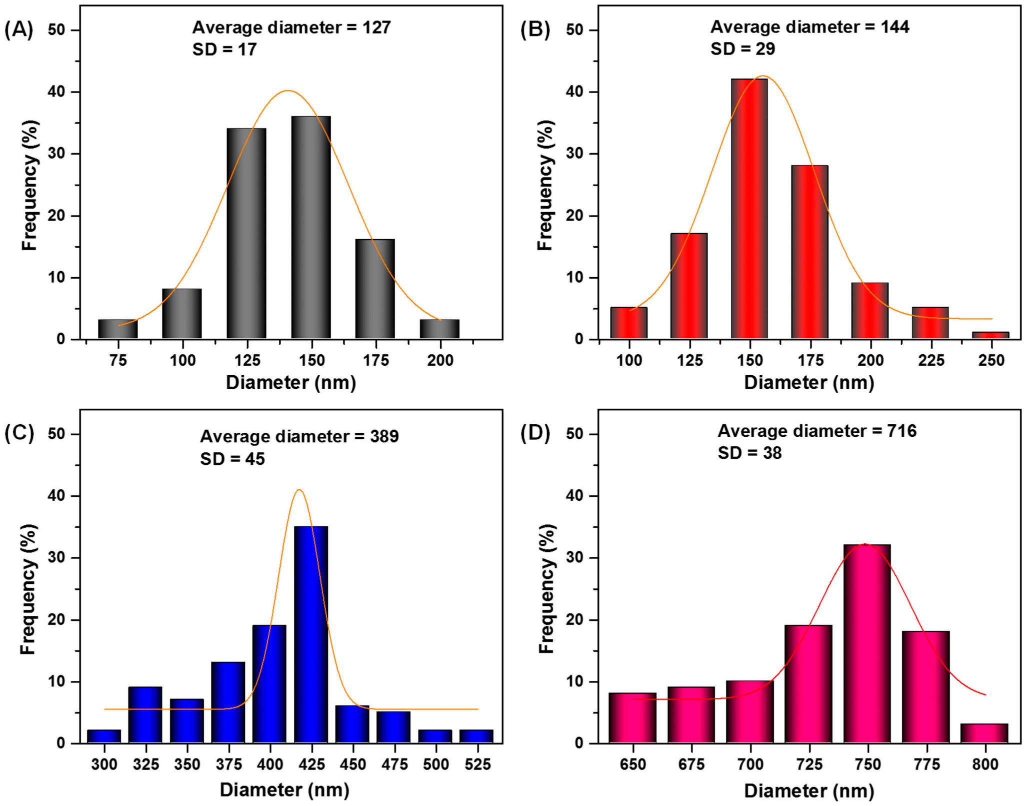

Figure S1. The diameter distributions of electrospun PAN NFMs of different concentration (**A)** 8 wt %, (**B**) 10 wt %, (**C**) 12 wt % and (**D**) 15 wt %.

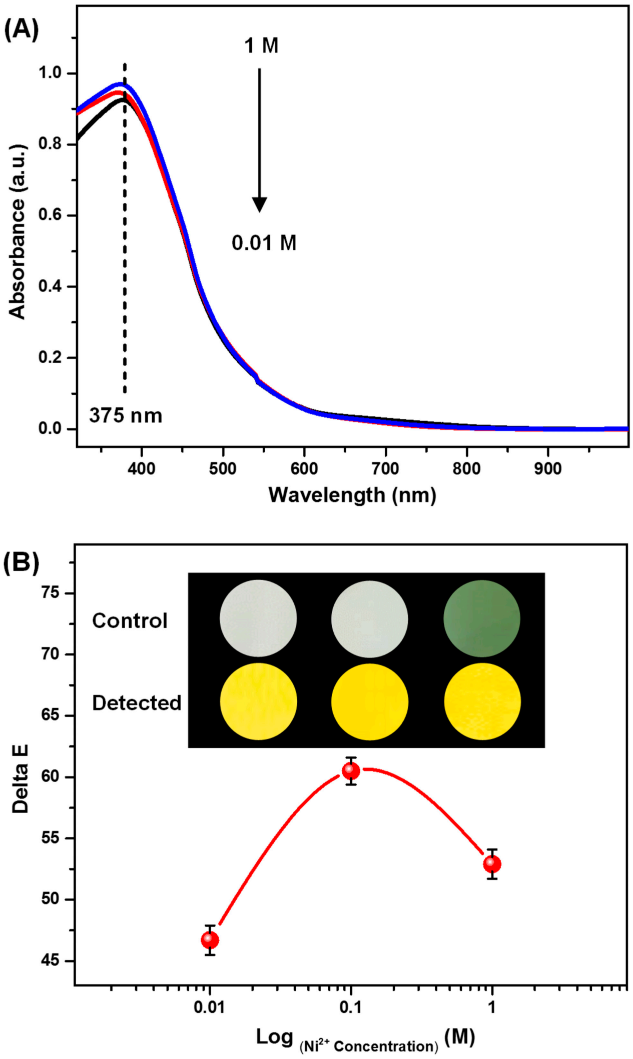

Figure S2. (**A**) The absorption spectra, and (**B**) the color difference values between the control and detected samples of the colorimetric strips as a function of Ni^{2+} concentration; the corresponding optical images are shown as inserts.

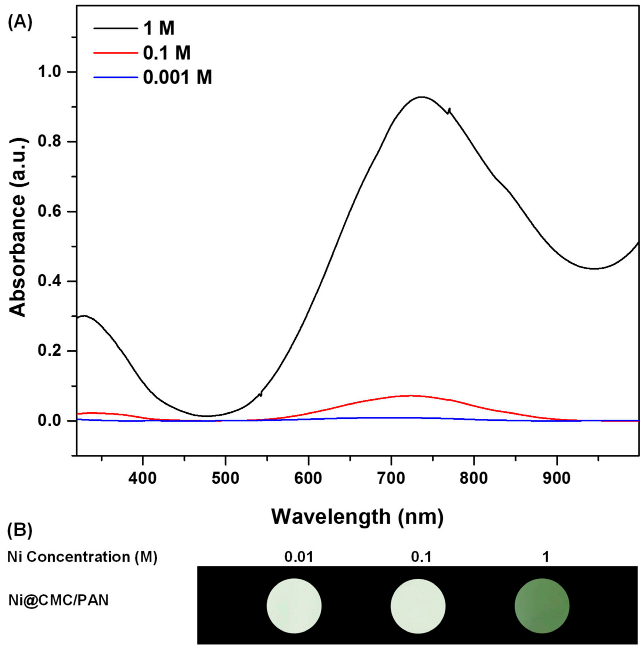

Figure S3. (**A**) The absorption spectra, and (**B**) the corresponding optical images of the CMC/PAN NFM upon exposure to Ni^{2+} solution at different concentrations (0.01, 0.1 and 1 M).

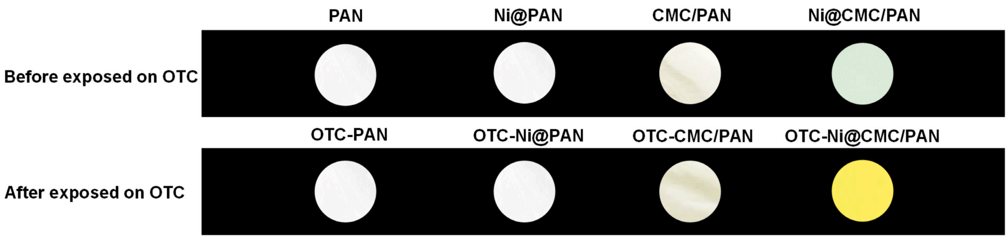

Figure S4. The color response of different NFM after exposure to 10 mM OTC solution for 30 min.

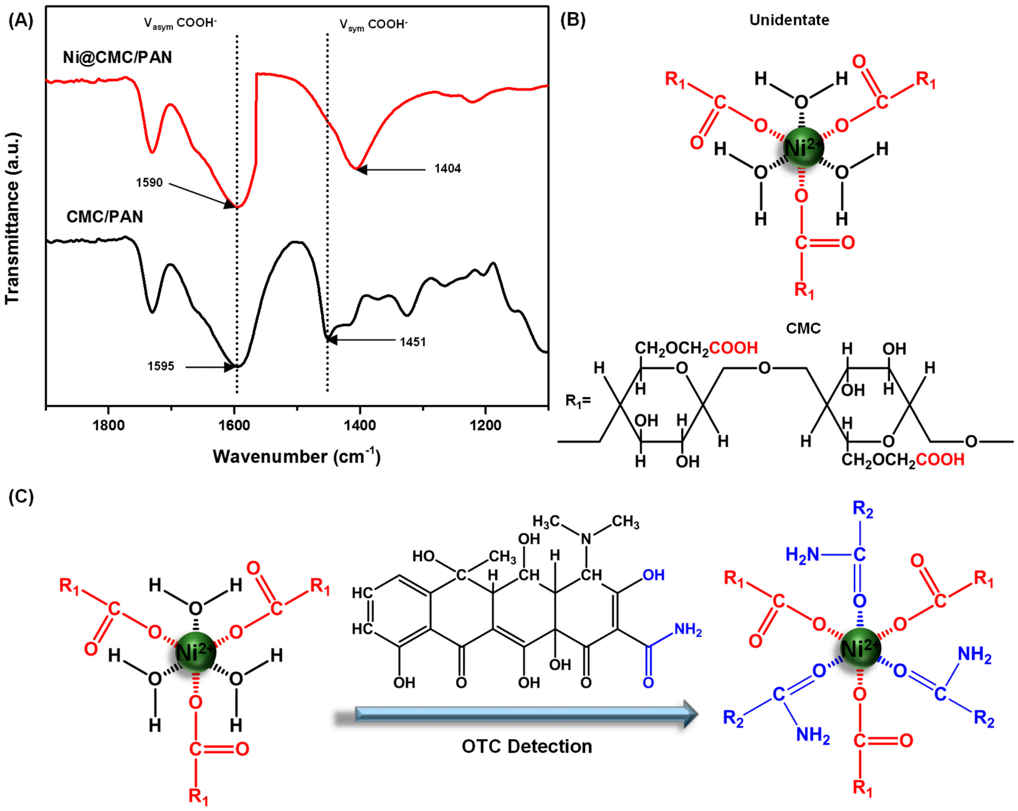

Figure S5. (**A**) FTIR spectra of CMC coated PAN FNM before and after Ni^{2+} immobilization and (**B**) the coordination mode of CMC with Ni^{2+}.

Table S1. The coating ratio of PAN NFM coated by 0.5 wt % CMC.

PAN Concentrations (wt %)	Weight before Coating (mg)	Weight after Coating (mg)	Coating Ratio (%)
8	11.5 ± 0.3	14.8 ± 0.4	28.3 ± 0.5
10	11.7 ± 0.3	14.9 ± 0.4	27.3 ± 0.3
12	11.8 ± 0.2	15.0 ± 0.2	26.7 ± 0.4
15	12.1 ± 0.2	15.1 ± 0.2	25.1 ± 0.3

Table S2. The Coating ratio of 8 wt% PAN NFM coated CMC with different concentration.

CMC Concentrations (wt %)	Weight before Coating (mg)	Weight after Coating (mg)	Coating Ratio (%)	Average Diameter (nm)
0.1	12.1 ± 0.2	12.7 ± 0.2	5.2 ± 0.1	131 ± 13
0.3	11.7 ± 0.3	13.6 ± 0.4	16.2 ± 0.3	133 ± 14
0.5	11.5 ± 0.3	14.8 ± 0.4	28.3 ± 0.1	135 ± 16
0.7	12.3 ± 0.2	20.2 ± 0.3	64.2 ± 0.3	137 ± 21
0.9	11.9 ± 0.2	23.4 ± 0.4	96.8 ± 0.2	148 ± 12
1.1	12.2 ± 0.2	25.5 ± 0.6	108.9 ± 0.1	152 ± 15

Table S3. BET surface areas and total pore volume of the PAN_8 NFMs coated with various CMC concentration (0, 0.1, 0.3, 0.5, 0.7, 0.9, and 1.1 wt %).

Samples	BET Specific Surface Area ($m^2·g^{-1}$)	Total Pore Volume ($cm^3·g^{-1}$)
PAN_8	11.390	0.02931
$CMC_{0.1}/PAN_8$	10.612	0.02018
$CMC_{0.3}/PAN_8$	8.093	0.01564
$CMC_{0.5}/PAN_8$	6.549	0.01262
$CMC_{0.7}/PAN_8$	4.688	0.01280
$CMC_{0.9}/PAN_8$	2.831	0.00782
$CMC_{1.1}/PAN_8$	1.479	0.00269
